# 15-Deoxy-Δ^12,14^-Prostaglandin J_2_ Inhibits Osteolytic Breast Cancer Bone Metastasis and Estrogen Deficiency-Induced Bone Loss

**DOI:** 10.1371/journal.pone.0122764

**Published:** 2015-04-10

**Authors:** Ki Rim Kim, Hyun Jeong Kim, Sun Kyoung Lee, Gwang Taek Ma, Kwang Kyun Park, Won Yoon Chung

**Affiliations:** 1 Department of Dental Hygiene, Kyungpook National University, Sangju, 742–711, Korea; 2 Department of Oral Biology, Oral Cancer Research Institute, BK21 PLUS project, Yonsei University College of Dentistry, Seoul, 120–752, Korea; 3 Department of Applied Life Science, The Graduate School, Yonsei University, Seoul, 120–749, Korea; Faculté de médecine de Nantes, FRANCE

## Abstract

Breast cancer is the major cause of cancer death in women worldwide. The most common site of metastasis is bone. Bone metastases obstruct the normal bone remodeling process and aberrantly enhance osteoclast-mediated bone resorption, which results in osteolytic lesions. 15-deoxy-Δ^12,14^-prostaglandin J_2_ (15d-PGJ_2_) is an endogenous ligand of peroxisome proliferator-activated receptor gamma (PPARγ) that has anti-inflammatory and antitumor activity at micromolar concentrations through PPARγ-dependent and/or PPARγ-independent pathways. We investigated the inhibitory activity of 15d-PGJ_2_ on the bone loss that is associated with breast cancer bone metastasis and estrogen deficiency caused by cancer treatment. 15d-PGJ_2_ dose-dependently inhibited viability, migration, invasion, and parathyroid hormone-related protein (PTHrP) production in MDA-MB-231 breast cancer cells. 15d-PGJ_2_ suppressed receptor activator of nuclear factor kappa-B ligand (RANKL) mRNA levels and normalized osteoprotegerin (OPG) mRNA levels in hFOB1.19 osteoblastic cells treated with culture medium from MDA-MB-231 cells or PTHrP, which decreased the RANKL/OPG ratio. 15d-PGJ_2_ blocked RANKL-induced osteoclastogenesis and inhibited the formation of resorption pits by decreasing the activities of cathepsin K and matrix metalloproteinases, which are secreted by mature osteoclasts. 15d-PGJ_2_ exerted its effects on breast cancer and bone cells via PPARγ-independent pathways. In Balb/c *nu/nu* mice that received an intracardiac injection of MDA-MB-231 cells, subcutaneously injected 15d-PGJ_2_ substantially decreased metastatic progression, cancer cell-mediated bone destruction in femora, tibiae, and mandibles, and serum PTHrP levels. 15d-PGJ_2_ prevented the destruction of femoral trabecular structures in estrogen-deprived ICR mice as measured by bone morphometric parameters and serum biochemical data. Therefore, 15d-PGJ_2_ may be beneficial for the prevention and treatment of breast cancer-associated bone diseases.

## Introduction

Breast cancer is inextricably linked to two bone diseases, bone metastasis and osteoporosis. Metastatic breast cancer cells in the bone microenvironment disturb the balance between osteoclasts and osteoblasts, which disrupts the bone remodeling cycle and results in bone destruction [1]. Therefore, a “vicious cycle” between tumor cells and the bone microenvironment plays a critical role in breast cancer-mediated bone loss [2–3]. Four essential contributors to this vicious cycle are tumor cells, osteoblasts, osteoclasts, and resorbed bone matrix. Tumor cells produce osteolytic factors, including parathyroid hormone-related protein (PTHrP) and several interleukins [4]. These factors stimulate the expression of receptor activator of nuclear factor-kappaB (RANK) ligand (RANKL) and inhibit the production of osteoprotegerin (OPG), which is a decoy receptor of RANKL, in osteoblastic/stromal cells. RANKL triggers osteoclast differentiation via binding to RANK on osteoclast precursors [5]. Bone resorption by mature osteoclasts releases calcium and growth factors, such as transforming growth factor-beta (TGF-β) and insulin-like growth factor-1, from the bone matrix. These growth factors further stimulate tumor growth and the secretion ofosteolytic factors from tumor cells, which causes severe osteolytic lesions [3,6]. In addition to the direct harm of bone metastasis, cancer therapy for early stage and/or estrogen receptor-positive breast cancer, including cytotoxic chemotherapy, induces premature ovarian failure and hormone deprivation therapy, which ultimately increases the risk of bone loss because of estrogen deficiency [7]. Therefore, the maintenance and restoration of bone health is particularly important to promote the efficacy of cancer treatment and the quality of life in breast cancer patients.

15-deoxy-Δ^12,14^-prostaglandin J_2_ (15d-PGJ_2_) is one of the terminal products of the cyclooxygenase-mediated arachidonic acid pathway, and it is an endogenous ligand of peroxisome proliferator-activated receptor gamma (PPARγ) [8]. Its cyclopentenone structure forms a covalent adduct with cysteine residues in protein targets, which contributes to its anti-inflammatory activity at micromolar concentrations [9]. Unlike pro-inflammatory prostaglandins, 15d-PGJ_2_ suppresses proliferation and induces apoptosis in different cancer cells [10–16]. 15d-PGJ_2_ inhibited the invasive capacities of MDA-MB-231 human breast cancer cells via by upregulating a tissue inhibitor of matrix metalloproteinase-1 and decreasing gelatinase activity in conditioned media [17]. However, 15d-PGJ_2_ increased the expression of matrix metalloproteinase (MMP)-1 and vascular endothelial growth factor to induce angiogenesis in MCF-7 breast cancer cells [18,19]. PPARγ activation by rosiglitazone induced bone loss by reducing osteoblast differentiation and activating osteoclast differentiation [20]. However, a recent study showed that rosiglitazone inhibited TNF-α-induced osteoclast differentiation and bone resorption [21]. Several studies also demonstrated the inhibitory effect of PPARγ agonists, including 15d-PGJ_2_, ciglitazone, and troglitazone, on osteoclast formation [22–24].

This study determined the inhibitory activity of 15d-PGJ_2_ on cancer-associated bone diseases. We examined the effect of 15d-PGJ_2_ on the viability, migration, invasion, and secretion of PTHrP in MDA-MB-231 metastatic human breast cancer cells, RANKL and OPG expression in hFOB1.19 osteoblastic cells, RANKL-induced osteoclastogenesis in mouse bone marrow macrophages, and bone resorption by mature osteoclasts. We further evaluated the effect of 15d-PGJ_2_ on bone loss in mice that received an intracardiac inoculation of human metastatic breast cancer cells and ovariectomized mice, which reflected estrogen deficiency.

## Materials and Methods

### Materials

15d-PGJ_2_ and the PPARγ antagonist GW9662 were purchased from Cayman Chemicals (Ann Arbor, MI), dissolved in dimethyl sulfoxide (DMSO), and diluted with culture media immediately prior to use. Dulbecco’s modified Eagle’s medium (DMEM), minimum essential medium-alpha (α-MEM), DMEM:nutrient mixture F-12 (DMEM/F-12) without phenol red, Dulbecco’s phosphate-buffered saline (PBS), Hanks’ balanced salt solution (HBSS), fetal bovine serum (FBS), an antibiotic-antimycotic mixture (100 U/ml penicillin and 100 μg/ml streptomycin), 0.25% trypsin-EDTA, and Geneticin (G-418) were products of Gibco-BRL (Grand Island, NY). Recombinant mouse soluble RANKL and murine macrophage-colony stimulating factor (M-CSF) were obtained from Koma Biotech (Seoul, South Korea) and R&D Systems (Minneapolis, MN), respectively. Zoledronic acid (di-sodium salt) was purchased from Enzo Life Sciences (Farmingdale, NY) and D-luciferin potassium salt was obtained from Goldbio Technology (St. Louis, MO). Histopaque-1083, 3-(4,5-dimethylthiazol-2-yl)-2,5-diphenyltetrazolium bromide (MTT), 17β-estradiol (E2), and DMSO were obtained from Sigma-Aldrich (St. Louis, MO). All reagents used in this study were of analytical grade.

### Cell culture

MDA-MB-231 human mammary carcinoma cells (Korean Cell Line Bank, Seoul, South Korea) were cultured in DMEM supplemented with 10% FBS and a 1% antibiotic-antimycotic mixture at 37°C under a humidified atmosphere of 5% CO_2_. Human fetal osteoblastic hFOB1.19 cells (American Type Culture Collection, Manassas, VA) were grown in DMEM/F-12 without phenol red, containing 10% FBS, 1% antibiotic-antimycotic mixture, and 0.3 mg/ml G418 at 34°C in 5% CO_2_ in a humidified incubator. Mouse bone marrow macrophages (BMMs) were isolated from the tibiae of male ICR mice using histopaque density gradient centrifugation as described previously [6] and cultured in α-MEM containing 10% FBS, 1% antibiotic-antimycotic mixture, and 30 ng/ml M-CSF at 37°C in a humidified atmosphere of 5% CO_2_.

### Animals

Male ICR mice (3 weeks old, 20 ± 3 g), female Balb/c *nu/nu* mice (5 weeks old, 20 ± 3 g), and female sham-operated and ovariectomized (OVX) ICR mice (8–9 weeks old, 28 ± 2 g) were obtained from Central Lab Animal (Seoul, South Korea). The mice were provided free access to a standard chow diet (Orient, Seongnam, Korea) and tap water *ad libitum* and housed under specific pathogen-free conditions with a 12-h light/dark cycle and a relative humidity of 50 ± 5% at 22 ± 2°C. The Institutional Animal Care and Use Committee of Department of Laboratory Animal Resources, Yonsei Biomedical Research Institute, Yonsei University College of Medicine approved all animal experiments.

### Luciferase vector construction and transfection

The firefly luciferase gene from *Photinus pyralis* was amplified using polymerase chain reaction (PCR)-based methods and a pTAL-Luc vector (Clontech Laboratories, Palo Alto, CA) followed by subcloning into the pLenti6/V5 Directional TOPO cloning vector in the ViraPower Lentiviral Expression System (Invitrogen, Carlsbad, CA) to generate lentiviral particles with lentiviral vector-based luciferase. The pLenti6/V5-Luc plasmid was subjected to DNA sequencing analysis to confirm successful construction. Lentivirus particles were produced using cotransfection of the 293FT producer cell line with the pLenti6/V5-Luc plasmid and ViraPower Packaging Mix. Cells were transduced using 2 x 10^7^ lentiviral particles in the transduction enhancer Polybrene at 10 μg/ml to establish luciferase-transfected MDA-MB-231 stable cells (MDA-MD-231/Luc^+^). Blasticidin (10 μg/ml) was added to select stably transduced cells. Blasticidin-resistant clones exhibited V5 epitope detection against an anti-V5 antibody on Western blot analysis and revealed the maximum level of luciferase activity in a microplate spectrofluorometer (Molecular Devices, Palo Alto, CA).

### Cell viability assay

MDA-MB-231 cells (1 x 10^4^ cells/well) were seeded into a 96-well plate with 10% FBS-DMEM. The cells were incubated in serum-free media with various concentrations of 15d-PGJ_2_ for 24 or 72 h. hFOB1.19 human osteoblastic cells (1x 10^4^ cells/well) were incubated in serum-free media with the indicated concentrations of 15d-PGJ_2_ for 6 h or 24 h. BMMs (5 x 10^4^ cells/well) were cultured in media with the indicated concentrations of 15d-PGJ_2_ in the presence of 10% FBS and 30 ng/ml M-CSF for 5 days. Cell viability was determined using the MTT assay as described previously [6].

### Cell migration assay

MDA-MB-231 cells were seeded in a 6-well plate and allowed to grow to 90% confluency. One artificial wound per well was scratched into monolayers using the narrow end of a sterile micropipette tip, and the wounded areas were photographed. Cells were incubated in serum-free DMEM with mitomycin (5 μg/ml) and various concentrations of 15d-PGJ_2_. The scratched areas were photographed again 40 h later at the identical location of the initial image. The width of the wounded cell monolayer was measured using ImageJ software, and the percentage of wound closure was derived using the following formula: (1–(current wound width/initial wound width)) × 100.

### Transwell invasion assay

A cell invasion assay was performed using a Transwell chamber (Corning, Cambridge, MA) that contained a polycarbonate membrane filter (6.5 mm diameter, 8 μm pore size). The bottom of the filter was coated with 0.1% (w/v) gelatin. Matrigel (BD Biosciences, San Jose, CA), which is a mixture of basement membrane extracellular matrix proteins, was diluted with DMEM to a final concentration of 1 mg/ml and applied to coat the membrane filter. MDA-MB-231 cell suspensions (2 x 10^4^ cells/100 μl) with various concentrations of 15d-PGJ_2_ were added to the inserts of each coated Transwell. The lower chamber contained 600 μl of media with 1% FBS and 15d-PGJ_2_. Transwell chambers were incubated for 24 h at 37°C. Cells were fixed with 70% methanol, and the membranes were stained with hematoxylin. Non-invading cells on the upper surface of the membrane were scraped with cotton swabs, and invading cells that remained on the bottom surface were mounted on slides. Four random fields for each membrane were captured, and cells in the captured fields were quantified under a Zeiss AXio imager microscope (Carl Zeiss AG, Göttingen, Germany).

### PTHrP assay

MDA-MB-231 cells were seeded at a density of 1 x 10^5^ cells into a 96-well plate and incubated to adhere overnight. Cells were treated with the indicated concentrations of 15d-PGJ_2_, TGF-β, and/or GW9662 for 24 h. The plate was centrifuged, and media were collected. PTHrP levels in the collected culture media were quantified using a human PTHrP enzyme-linked immunosorbent assay (ELISA) kit (USCN Life Science, Wuhan, China) [25].

### Western blot analysis

MDA-MB-231 cells were seeded at 1 x 10^6^ cells in 100 mm cell culture dishes and incubated with the indicated concentrations of TGF-β, 15d-PGJ_2_, and/or GW9662 for 24 h. Cells were lysed in RIPA buffer (Cell Signaling Technology, Danvers, MA) containing 1 mM phenylmethylsulfonyl fluoride and protease inhibitor cocktail tablets (Roche Diagnostics) or the nuclear/cytosol fractionation kit (BioVision, Mountain View, CA). Samples were centrifuged, and proteins in the supernatants were quantitated using BCA protein assay reagents (Pierce Biotechnology, Rockford, IL). Proteins were separated using 12% sodium dodecyl sulfate-polyacrylamide gel electrophoresis and transferred to polyvinylidene difluoride membranes (Millipore, Danvers, MA). Membranes were blocked for 1 h in 5% skim milk and incubated with rabbit anti-Smad2, rabbit anti-phosphorylated Smad2 (Cell Signaling Technology), rabbit anti-β -actin (Sigma-Aldrich), and mouse anti-Lamin B (Invitrogen) at 4°C overnight, followed by incubation with horseradish peroxidase-conjugated secondary antibody for 1 h at room temperature. Proteins were visualized using an enhanced chemiluminescence kit (GE Healthcare, Buckinghamshire, UK).

### Quantitative real-time PCR

The medium was replaced with serum-free DMEM/F-12 when seeded MDA-MB-231 cells reached 70–80% confluency in 10% FBS-DMEM. Conditioned media (CM), including MDA-MB-231 cell-secreted osteolytic factors, were collected after a 24-h incubation. Osteoblastic hFOB1.19 cells (1 x 10^6^ cells/100 mm culture dish) were treated with the indicated concentrations of 15d-PGJ_2_ and/or GW9662 in DMEM with 75% CM or PTHrP (100 nM) for 6 h. Total RNA was isolated using an RNeasy Mini Kit (Qiagen, Valencia, CA). First-strand cDNA from 1 μg total RNA was synthesized using the PrimeScript RT reagent kit (TaKaRa, Dalian, China). Real-time quantitative RT-PCR was performed using the 7300 Real-Time PCR System (Applied Biosystems, Foster City, CA) and the SYBR Premix Ex Taq (TaKaRa) in a 96-well optical reaction plate according to the manufacturer’s instructions. The following PCR conditions were used: initial denaturation at 95°C for 30 s, followed by 40 cycles of denaturation at 95°C for 5 s and annealing at 60°C for 31 s. Cycle threshold (Ct) values were established. Relative gene expressions of RANKL and OPG to the reference gene GAPDH were determined using the 2^−ΔΔCt^ method. The following primer sequences were used: RANKL, forward 5’-ATGGTGGATGGCTCATGGTTAG-3’ and reverse 5’-GAGCAAAAGGCTGAGCTTCAAG-3’; OPG, forward 5′-CCAGTGACCAGATCCTGAAGCT-3′ and reverse 5′-GGTGTCTTGGTCGCCATTTT-3′; and GAPDH, forward 5′-AGTCCTTCCACGATACCAAAGT-3′ and reverse 5′-CATGAGAAGTATGACAACAGCCT-3′.

### Osteoclast formation assay

BMMs (5 x 10^4^ cells/well) were seeded into a 96-well plate and cultured in α-MEM with 10% FBS, 30 ng/ml M-CSF and the indicated concentrations of 15d-PGJ_2_ in the absence or presence of 100 ng/ml RANKL. Cells were cultured for 5 days, and fresh media containing the appropriate chemicals was replaced every other day. Cells were fixed using 3.7% (v/v) formaldehyde and stained for tartrate-resistant acid phosphatase (TRAP) activity for 10 min at 37°C using the Acid Phosphatase Leukocyte kit (Sigma-Aldrich) as described previously [6,26]. TRAP-positive multinucleated cells (MNCs) with more than three nuclei were quantified as differentiated osteoclasts using an Olympus IX70 inverted microscope (Olympus Optical, Tokyo, Japan) (100x magnification).

### Pit formation assay

BMMs (5 x 10^4^ cells/well) were plated onto BD BioCoat Osteologic MultiTest Slides with mineralized calcium phosphate thin films (BD Biosciences) and cultured in α-MEM containing 10% FBS, 30 ng/ml M-CSF, and 100 ng/ml RANKL for 4 days as described previously [26]. Differentiated osteoclasts were treated with 15d-PGJ_2_ at the indicated concentrations for an additional 10 days. Media were replaced every other day. Media were collected to measure cathepsin K and MMP activity, and cells were treated with 4% sodium hypochlorite. Slides were washed twice using distilled water, and resorbed pits were observed under a light microscope (100x magnification).

### Cathepsin K activity assay and gelatin zymography

The activity of cathepsin K and MMPs in the collected media was measured using a SensiZyme Cathepsin K Activity Assay Kit (Sigma-Aldrich) and gelatin zymography as described previously [26]. Cathepsin K activity was calculated using a standard curve and the gelatinolytic activities of MMPs were detected as clear bands against a dark blue background.

### Animal model of breast cancer bone metastasis

MDA-MB-231/Luc^+^ cells (1 x 10^6^ cells/0.1 ml in HBSS) were injected into the left cardiac ventricle of female Balb/c *nu/nu* mice as previously described [27,28]. Animals were divided into four groups of 10 mice on the following day and subcutaneously administered vehicle (PBS containing 2% DMSO) alone, 0.5 or 2 mg/kg 15d-PGJ_2_, or 0.1 mg/kg zoledronic acid as a positive control, three times per week for six weeks. Metastatic progression in nude mice was visualized using bioluminescence imaging three and five weeks after intracardiac injections. Mice were anesthetized for imaging using a Xenogen XGI-8 Gas Anesthesia System and injected intraperitoneally with 150 mg/kg of D-luciferin potassium salt in PBS. The luciferase activity was visualized using an intensified CCD video camera connected to the *in vivo* IVIS Imaging System 200 Series (Caliper Life Sciences, Hopkinton, MA). Bioluminescence from mice was expressed as total photon flux measured in photons/sec/cm^2^ per steradian (sr) using Xenogen Living Image software. Blood was collected by intracardiac puncture at the end of the experiment for the serum PTHrP assay. The femora, tibiae, and mandibles of nude mice were also collected for μCT analysis.

### A murine model of ovariectomy-induced bone loss

OVX mice were divided into four groups of 10 mice, and subcutaneously administered vehicle (PBS containing 2% DMSO), 0.5 or 2 mg/kg 15d-PGJ_2_, or 10 μg/kg E2 as a positive control, three times per week for 10 weeks. Sham-operated mice were treated with vehicle alone. Body weights were measured weekly using an electronic scale. Blood samples were collected by cardiac puncture at the end of the experimental period, and the femora were dissected.

### μCT analysis

The femora, tibiae, and mandibles of nude mice and the femora of sham-operated and OVX mice were examined using a SkyScan 1076 μCT scanner (SkyScan, Aartselaar, Belgium) with 100 kV, 140 μA current, rotation step 0.6°, and camera pixel size of 35 μm as described previously [6,26]. Three-dimensional (3D) images were reconstructed based on the μCT images using SkyScan NRecon software and analyzed using SkyScan's computed tomography analyzer software (CTAn). The following parameters were analyzed in the proximal tibiae of nude mice and the distal femora of sham-operated and OVX mice for quantitative analyses of bone histomorphometry: percent bone volume (BV/TV, %), trabecular thickness (Tb.Th, mm), trabecular number (Tb.N, mm^-1^), trabecular separation (Tb.Sp, mm), and structure model index (SMI). Values for bone mineral density (BMD) were measured in the femora of sham-operated and OVX mice.

### Determination of biochemical bone parameters

The collected blood samples were allowed to clot for 2 h at room temperature and centrifuged at 2,000 *×g* for 20 min to obtain sera as described previously [6,26]. Serum PTHrP levels from 10 nude mice were quantified in triplicate using a specific PTHrP ELISA kit. Calcium and alkaline phosphatase (ALP) levels in the sera of sham-operated and OVX mice were determined using QuantiChrome calcium and ALP assay kits (BioAssay Systems, Hayward, CA), respectively. TRAP and C-terminal telopeptides of type I collagen (CTX) levels were measured using mouse TRAP and RatLaps enzyme immunoassay (EIA) kits (Immunodiagnostic Systems, Fountain Hills, AZ), respectively. Serum levels of osteocalcin were detected using a mouse osteocalcin EIA kit (Biomedical Technologies, Stoughton, MA), and tumor necrosis factor-alpha (TNF-α) and interleukin-1 beta (IL-1β) levels were quantified using respective commercially available ELISA kits (R&D Systems). The serum levels of these factors in 10 sham-operated and OVX mice were measured in duplicate.

### Goldner's trichrome staining

Mouse femora were fixed in a 10% buffered formalin solution for 48 h, decalcified with a 10% EDTA solution, and embedded in paraffin. Five-μm–thick serial sagittal sections were stained using Goldner's trichrome according to the protocol specified by Electron Microscopy Sciences (Hatfield, PA).

### Statistical analysis

Data are expressed as the means ± standard error (SEM) and analyzed using one-way ANOVA and Student’s *t*-test. A value of *P*< 0.05 was considered statistically significant.

## Results

### 15d-PGJ_2_ reduced the viability, migration, and invasion of MDA-MB-231 breast cancer cells

**Fig 1:**
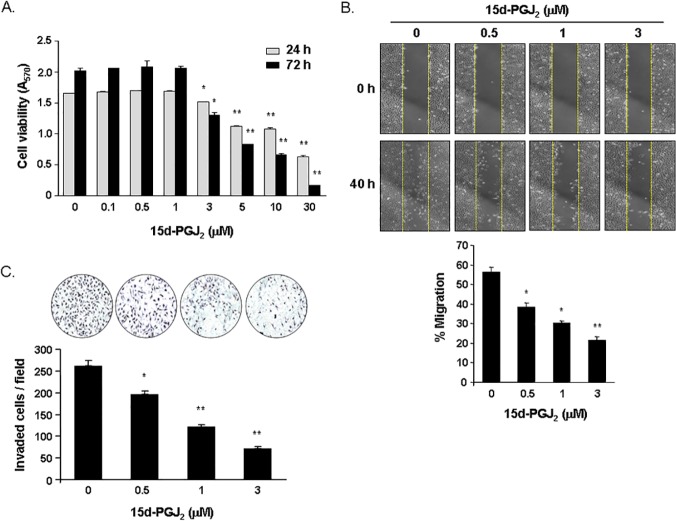
Effect of 15d-PGJ_2_ on the viability, migration, and invasion of MDA-MB-231 cells. (A) Cells were incubated in serum-free media containing various concentrations of 15d-PGJ_2_ for 24 or 72 h. Cell viability was determined using the MTT assay. (B) Cells were grown to confluency in monolayers, scratched using a micropipette tip, and treated with the indicated concentrations of 15d-PGJ_2_ for 40 h. Scratched areas on cultured MDA-MB-231 cells were observed under a light microscope immediately and 40 h after scratching (40x magnification). Relative migrating distances of cells into scratched areas were measured using ImageJ software. Data are expressed as percentages of cell migrating distances at 40 h compared with 0 h. (C) Cells were stimulated with a 1% FBS attractant and treated with 15d-PGJ_2_ at the indicated concentrations for 24 h. Cells that traversed across the Matrigel matrix were stained with hematoxylin, and representative images were visualized using light microscopy (200x magnification). The numbers of invaded cells were counted in four random fields per membrane filter. Data are expressed as means ± SEM. *P<0.05, **P<0.001 vs. untreated cells.

The viability of MDA-MB-231 cells treated with the indicated concentrations of 15d-PGJ_2_ for 24 or 72 h was reduced in a dose-dependent manner (Fig 1A). Cells exposed to 3, 5, 10, and 30 μM concentrations of 15d-PGJ_2_ were viable to 91%, 65%, 54%, and 38%, respectively, after 24 h of treatment and 65%, 33%, 22%, and 8%, respectively, after 72 h of treatment. The scratch wound healing assay showed that a 40 h treatment with 15d-PGJ_2_ at noncytotoxic concentrations inhibited the migratory ability of MDA-MB-231 cells by 32% at 0.5 μM, 46% at 1 μM, and 62% at 3 μM (Fig 1B). 15d-PGJ_2_ also decreased cell invasion in a dose-dependent manner. The number of invaded cells was inhibited by 25%, 53%, and 73% following treatment for 24 h with 15d-PGJ_2_ at concentrations of 0.5, 1, and 3 μM, respectively (Fig 1C).

### 15d-PGJ_2_ inhibited PTHrP production in MDA-MB-231 breast cancer cells

**Fig 2:**
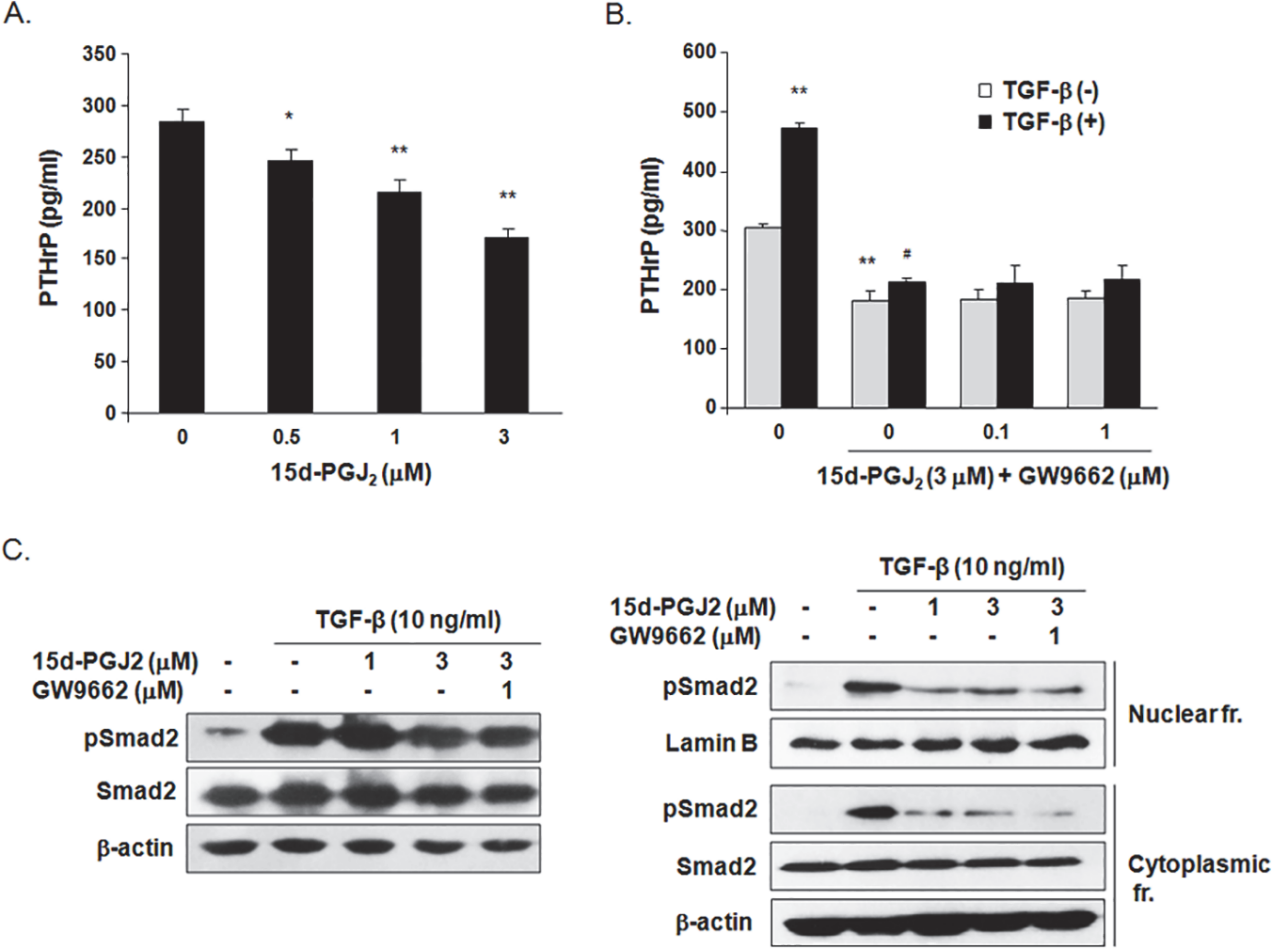
Effect of 15d-PGJ_2_ on PTHrP production in MDA-MB-231 cells. The cells were treated with (A) various concentrations of 15d-PGJ_2_ or (B) TGF-β, 15d-PGJ_2_ and/or GW9662 for 24 h. PTHrP levels were measured in the cultured media of MDA-MB-231 cells using a commercial human PTHrP ELISA kit. Data are expressed as the means ± SEM. *P<0.05, **P<0.01 vs. untreated cells. ^#^ *P*<0.01 vs. TGF-β -treated cells. (C) The level of Smad2 in total lysates and nuclear and cytoplasmic fractions was determined using western blotting in MDA-MB-231 cells stimulated by TGF- β, 15d-PGJ_2_ and/or GW9662 PPARγ antagonist for 24 h. The cropped blots are representative of experiments that were repeated three times.

We investigated the effect of 15d-PGJ_2_ on the secretion of PTHrP, which plays a central role in the osteolytic bone metastasis of MDA-MB-231 cells [29]. PTHrP levels were decreased by 24% in the culture media of MDA-MB-231 cells treated with 1 μM 15d-PGJ_2_ for 24 h and 39% with 3 μM 15d-PGJ_2_ (Fig 2A). TGF-β stimulation elevated PTHrP levels in the culture media by 1.6-fold, but treatment with 3 μM 15d-PGJ_2_ noticeably inhibited TGF-β-induced PTHrP production in MDA-MB-231 cells. Treatment with GW9662, a PPAR-γ antagonist, did not normalize the reduced PTHrP levels following 15d-PGJ_2_ treatment in MDA-MB-231 cells regardless of TGF-β treatment (Fig 2B). 15d-PGJ_2_ treatment inhibited the substantial enhancement in Smad2 phosphorylation and nuclear levels of pSmad2 in TGF-β -stimulated cells. The inhibitory effect of 15d-PGJ_2_ on TGF-β -induced activation of Smad2 remained in the presence of GW9662 (Fig 2C). These results demonstrate that 15d-PGJ_2_ inhibits PTHrP production through a PPARγ-independent pathway in MDA-MB-231 breast cancer cells regardless of TGF- stimulation.

### 15d-PGJ_2_ inhibited the RANKL/OPG ratio in hFOB1.19 osteoblastic cells

**Fig 3:**
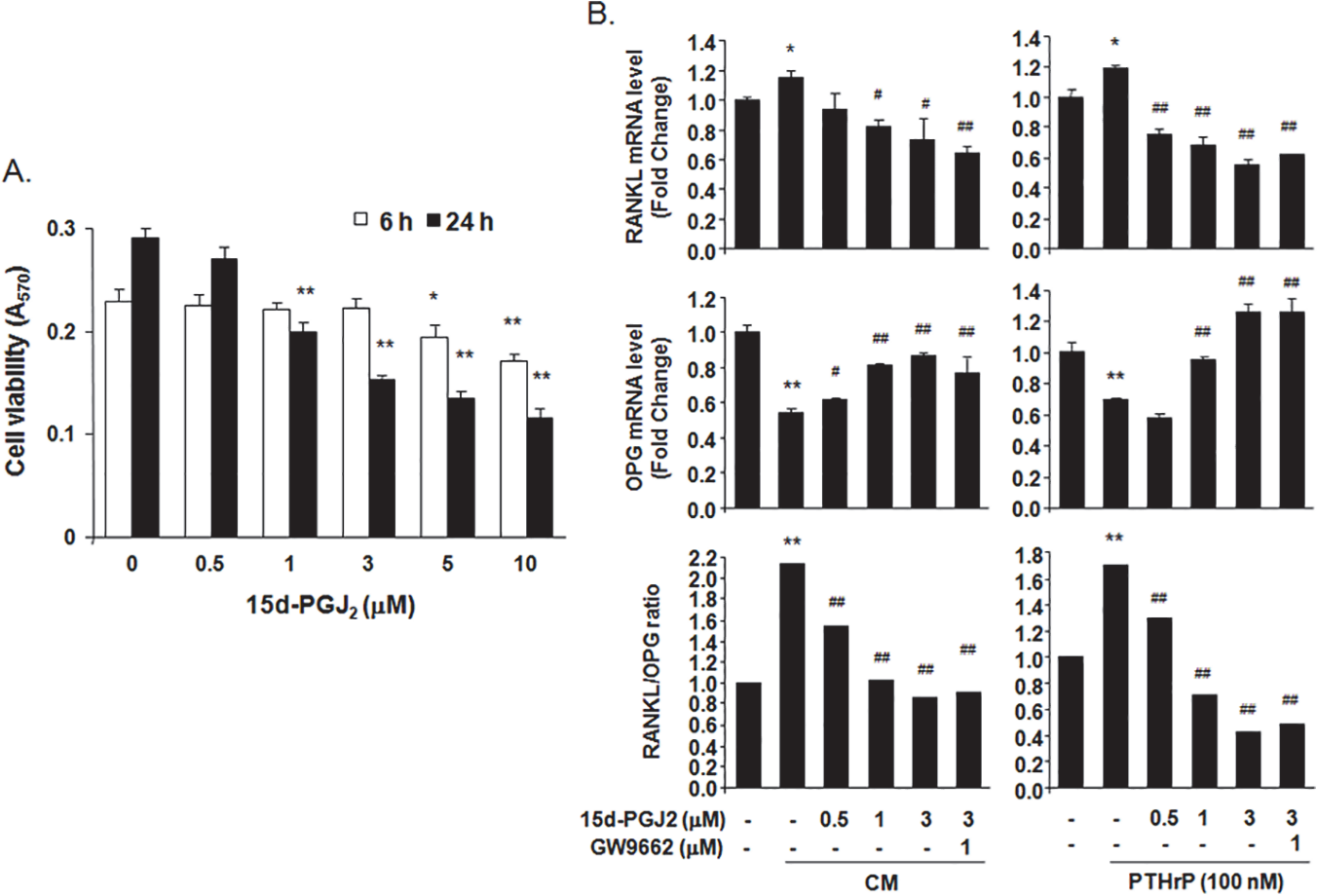
Effect of 15d-PGJ_2_ on RANKL and OPG mRNA expression in hFOB1.19 human osteoblastic cells. (A) hFOB1.19 cells were treated with the indicated concentrations of 15d-PGJ_2_ in DMEM/F12 for 6 h or 24 h. Cell viability was determined using the MTT assay. (B) hFOB1.19 cells were treated with the indicated concentrations of 15d-PGJ_2_ in DMEM/F12 containing 75% CM of MDA-MB-231 cells or PTHrP (100 ng) for 6 h. mRNA levels of RANKL and OPG were analyzed using real time-PCR. Graphs are expressed as the ratio of the densitometric intensity of RANKL to OPG after normalization to GAPDH. Data represent the means ± SEM. *P<0.05, **P<0.001 vs. untreated cells, ^#^ *P*<0.05, ^##^ *P*<0.001 vs. CM- or PTHrP-treated cells.

15d-PGJ_2_ treatment at concentrations less than 5 μM for 6 h did not show cytotoxic effects, but a 24-h treatment inhibited cell viability by 47% at 3 μM and 60% at 10 μM in hFOB1.19 human osteoblastic cells (Fig 3A). Real-time PCR analysis indicated that RANKL mRNA levels increased considerably and OPG mRNA levels decreased noticeably in osteoblastic hFOB1.19 cells stimulated with 75% CM from MDA-MB-231 breast cancer cells or PTHrP (100 nM) for 6 h, which elevated the RANKL/OPG ratio. However, treatment with 15d-PGJ_2_ dose-dependently suppressed the CM- and PTHrP-induced increase in the RANKL/OPG ratio by blocking increases in RANKL mRNA levels and decreases in OPG mRNA levels in hFOB1.19 cells exposed to CM and PTHrP, respectively (Fig 3B). GW9662 treatment did not rescue RANKL and OPG mRNA levels that were altered by 15d-PGJ_2_ treatment in CM and PTHrP-treated hFOB1.19 cells. These results indicate that 15d-PGJ_2_ inhibits the RANKL/OPG ratio in hFOB1.19 cells stimulated with MDA-MB-231 cell-derived osteolytic factors, particularly PTHrP.

### 15d-PGJ_2_ inhibited RANKL-induced osteoclast differentiation and activity

**Fig 4:**
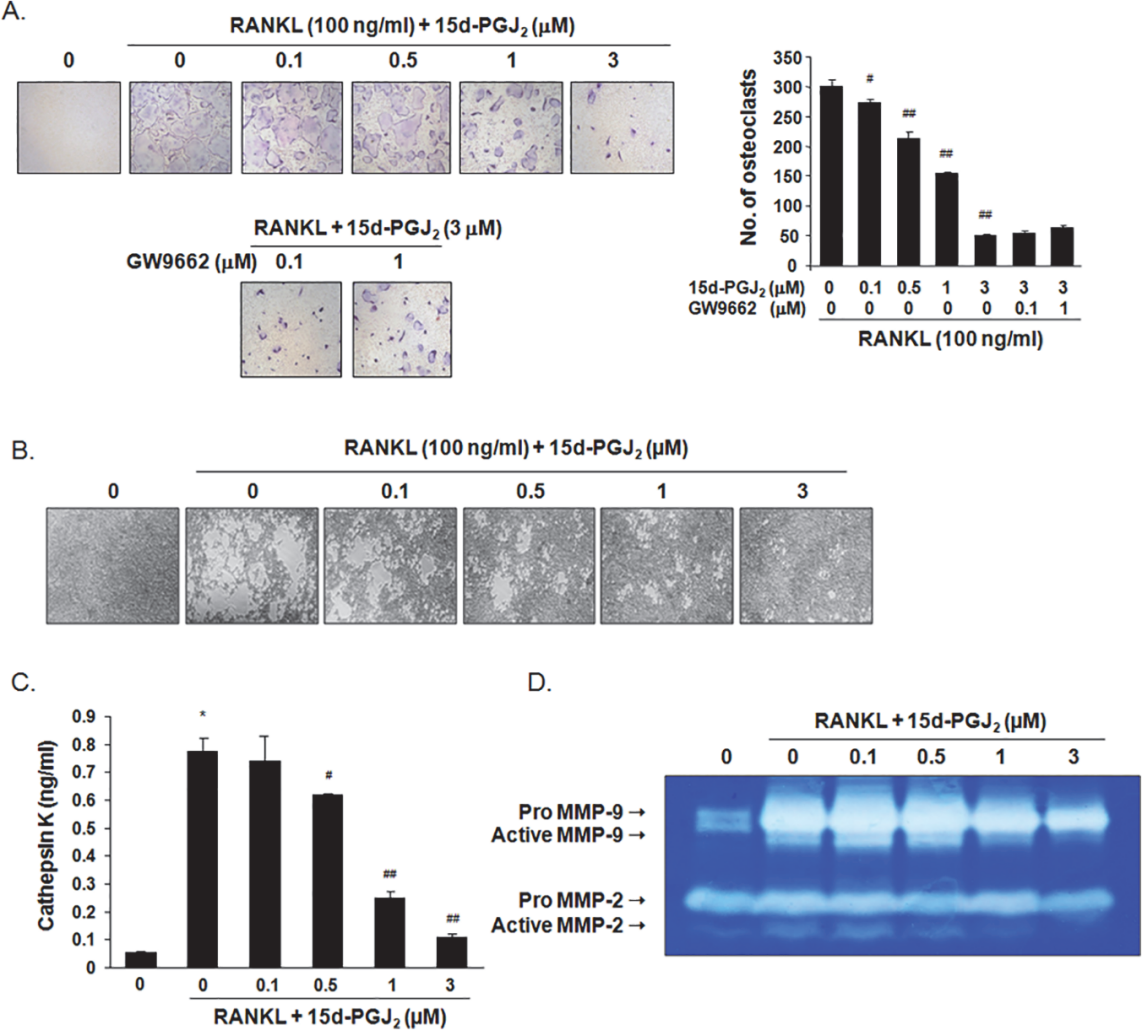
Effect of 15d-PGJ_2_ on RANKL-induced osteoclast differentiation and activation. (A) BMMs isolated from ICR mice were treated with M-CSF (30 ng/ml), RANKL (100 ng/ml), 15d-PGJ_2_, and/or GW9662 for 5 days. TRAP staining was performed to detect osteoclast formation. TRAP-positive multinucleated cells (≥ 3 nuclei) as differentiated osteoclasts were observed (100x magnification) and counted under an inverted microscope. (B) The differentiated BMMs were treated with 15d-PGJ_2_ in the presence of M-CSF (30 ng/ml) and RANKL (100 ng/ml) for an additional 10 days. The formed resorption pits were visualized using light microscopy (100x magnification). (C) The level of cathepsin K in the cultured media was measured using a commercially available ELISA kit. (D) The activities of MMPs were determined using gelatin zymograpy as clear bands against a blue background that corresponded to active MMP-2/9 (62/92 kDa) and pro-MMP-2/9 (72/105 kDa). The cropped gel image is representative of experiments that were repeated three times. Data are expressed as the means ± SEM. *P<0.001 vs. RANKL-untreated cells. ^#^ *P*<0.05, ^##^ *P*<0.001 vs. RANKL-treated cells.

Treatment with 15d-PGJ_2_ for 5 days inhibited cell viability by 19% at a concentration of 3 μM, 36% at 5 μM, and 53% at 10 μM in BMMs. BMMs were differentiated to TRAP-positive multinucleated cells as osteoclasts in the presence of M-CSF and RANKL for 5 days, but treatment with 15d-PGJ_2_ inhibited RANKL-induced osteoclast formation in a dose-related manner. Treatment with 0.5, 1, and 3 μM 15d-PGJ_2_ reduced the number of differentiated osteoclasts by 32%, 55%, and 93%, respectively. GW9662 treatment did not attenuate the inhibitory effect of 15d-PGJ_2_ on RANKL-induced osteoclast formation (Fig 4A). The formation of resorption pits determined the activity of mature osteoclasts. Osteoclast differentiation was induced on calcium phosphate-coated plates, and cells were incubated with 15d-PGJ_2_ in the presence of M-CSF and RANKL for 10 days. Treatment with 15d-PGJ_2_ dose-dependently suppressed the formation of resorbed areas (Fig 4B). RANKL treatment significantly increased the activity of cathepsin K in cultured media, but 15d-PGJ_2_ treatment suppressed the activity almost to control levels (Fig 4C). Gelatin zymography indicated that the levels of pro- and active forms of MMP-2 and MMP-9 were considerably enhanced in cultured media of RANKL-induced mature osteoclasts, but 15d-PGJ_2_ treatment decreased the levels of these MMPs in a dose-dependent manner (Fig 4D).

### 15d-PGJ_2_ inhibited osteolytic bone metastasis by breast cancer cells in nude mice

**Fig 5:**
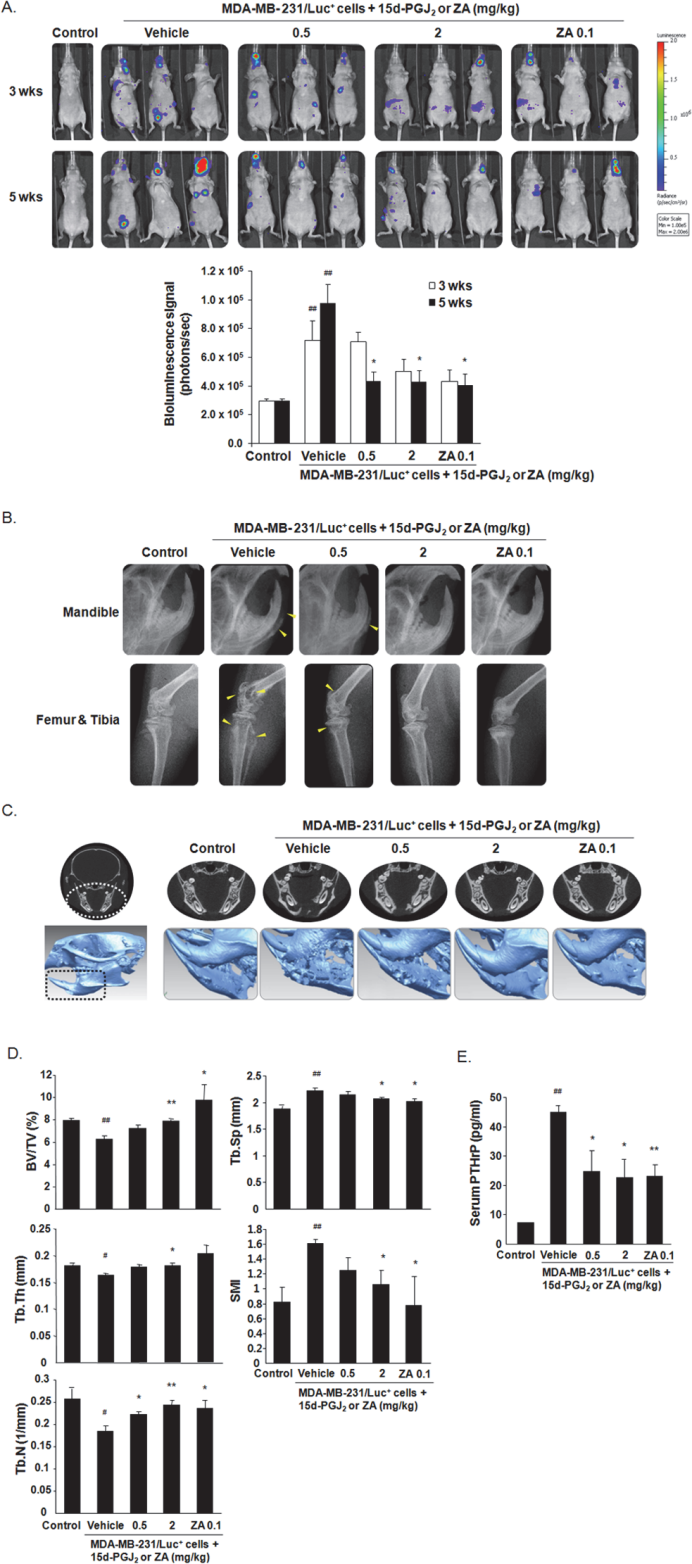
Effect of 15d-PGJ_2_ on osteolytic bone metastasis in nude mice that received intracardiac injections of MDA-MB-231 cells. MDA-MB-231/Luc^+^ cells were inoculated into the left ventricles of female nude mice. 15d-PGJ_2_ or zoledronic acid (ZA) was subcutaneously injected 3 times per week for 6 weeks at the indicated doses (*n* = 10). (A) Metastatic progression was detected by measuring bioluminescence in the same mice at 3 and 5 weeks after the injection of cancer cells. The formed metastases were quantified by measuring total photon flux per second. (B) Radiographic images of mandibles, distal femora, and proximal tibiae were scanned using μCT 6 weeks after the injection of cancer cells. Arrowheads indicate osteolytic lesions. (C) The mandibles of mice were analyzed using 3D-images. (D) Bone morphometric parameters, including BV/TV, Tb.N, Tb.Th, Tb.Sp, and SMI, were measured using μCT analysis of the proximal tibiae from mice. (E) Serum PTHrP levels were assayed using a commercially available ELISA kit. Data are expressed as the means ± SEM. ^#^ *P*<0.05, ^##^ *P*<0.01 vs. control group.*P<0.05, **P<0.01 vs. vehicle-treated group inoculated with cancer cells.

MDA-MB-231/Luc^+^ cells were inoculated into the left ventricles of nude mice to induce bone metastasis of breast cancer cells, and 15d-PGJ_2_ or zoledronic acid was subcutaneously injected for six weeks. Bioluminescence imaging showed that subcutaneous administration of 15d-PGJ_2_ or zoledronic acid for three and five weeks inhibited the metastatic progression of MDA-MB-231/Luc^+^ cells (Fig 5A). Osteolytic lesions of all mice were analyzed using μCT six weeks after cancer cell injections. Radiographic images of MDA-MB-231/Luc^+^ cell-injected mice indicated that osteolytic lesions were substantially developed in mandibles, distal femora, and proximal tibiae. However, 15d-PGJ_2_ or zoledronic acid administration inhibited cancer cell-induced osteolytic lesions in a dose-dependent manner (Fig 5B). 3D-images also revealed that MDA-MB-231/Luc^+^ cells induced severe destruction in the inner part of the mandible, but treatment with 15d-PGJ_2_ or zoledronic acid noticeably reduced this damage (Fig 5C). Bone morphometric analyses demonstrated that the injection of MDA-MB-231/Luc^+^ cells decreased BV/TV, Tb.Th, and Tb.N and increased Tb.Sp and SMI, but treatment with 15d-PGJ_2_ or zoledronic acid induced a recovery of these bone morphometric parameters. Furthermore, the levels of PTHrP were significantly increased in the sera of mice that received an intracardiac injection of MDA-MB-231/Luc^+^ cells, but treatment with 15d-PGJ_2_ or zoledronic acid inhibited the increase in PTHrP levels (Fig 5D).

### 15d-PGJ_2_ inhibited ovariectomy-induced bone loss

**Fig 6:**
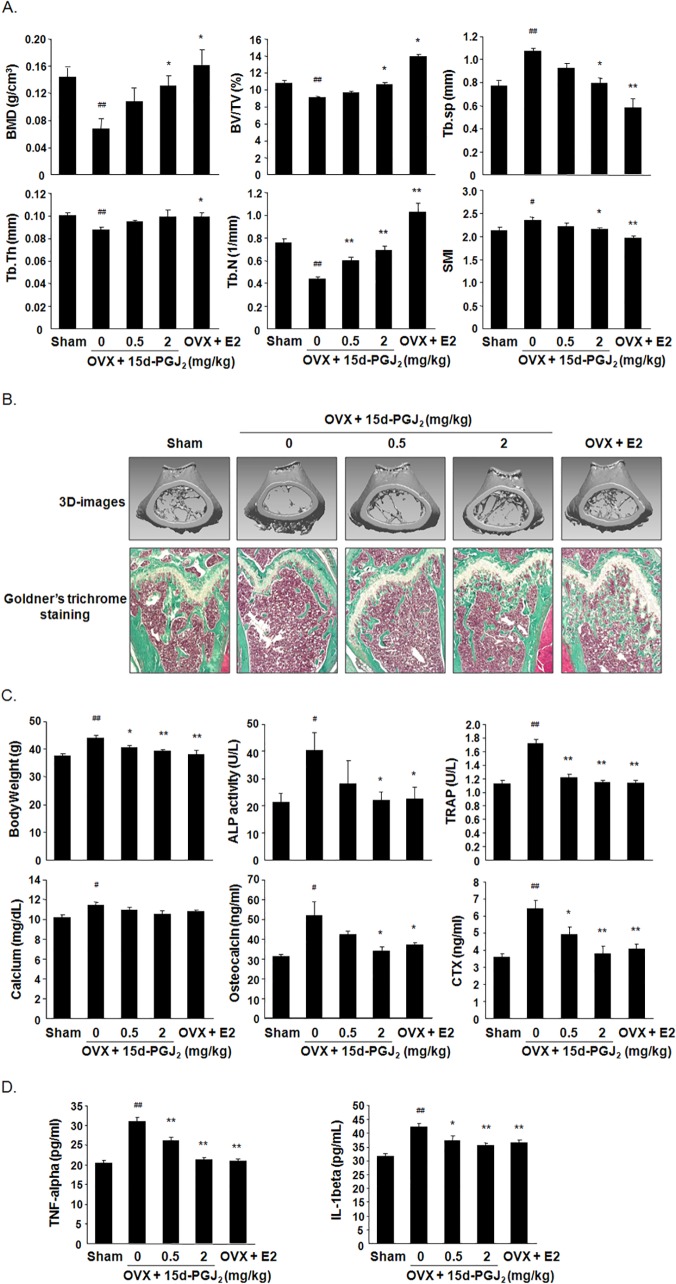
Effect of 15d-PGJ_2_ on ovariectomy-induced bone loss. OVX mice were subcutaneously injected with vehicle, 15d-PGJ_2_, or E2 (10 μg/kg) for 10 weeks (*n* = 10). Sham-operated mice received vehicle alone (*n* = 10). (A) Bone morphometric parameters, including BMD, BV/TV, Tb.Th, Tb.N, Th.Sp, and SMI, were measured using μCT analysis of the femora from mice. (B) 3D images of distal femora of mice were obtained from the reconstruction of μCT data (upper). Sagittal sections of distal femora from mice were stained with Goldner's trichrome. Bone trabeculae appear green, and bone marrow appears red. Stained sections were photographed using a light microscope (100x magnification). (C) Body weights of all mice were measured, and blood sera were collected from all mice for analyses of biochemical parameters. Serum levels of calcium, ALP, osteocalcin, TRAP, and CTX were evaluated using the respective kits as described in Materials and Methods. (D) Serum levels of TNF-α and IL-1β were determined using specific ELISA kits. Data are expressed as the means ± SEM. ^#^ *P*< 0.05, ^##^ *P*< 0.01 vs. sham group. *P<0.05, **P<0.01 vs. OVX group.

Ovariectomy-induced osteoporosis is commonly used as an animal model of bone loss due to estrogen deficiency in humans [30]. μCT analysis showed that BMD, BV/TV, Tb.Th and Tb.N decreased, but Tb.Sp and SMI increased, in OVX mice compared to the sham group. However, these bone morphometric parameters significantly recovered to control levels in 15d-PGJ_2_- or E2-treated OVX mice (Fig 6A). 3D-images and Goldner’s trichrome staining of distal femoral metaphyses revealed that the subcutaneous administration of 15d-PGJ_2_ or E2 in OVX mice dose-dependently suppressed severe trabecular bone loss in OVX mice (Fig 6B). In addition, treatment with 15d-PGJ_2_ or E2 blocked elevations in body weight in OVX mice. We measured ALP activity and osteocalcin level as markers of bone formation and calcium, TRAP, and CTX were used as the markers of bone resorption. The serum levels of these bone turnover markers were elevated significantly in OVX mice, but these levels noticeably decreased in 15d-PGJ_2_- or E2-treated OVX mice (Fig 6C). Treatment with 15d-PGJ_2_ or E2 also significantly reduced TNF-α and IL-1β levels in the sera of OVX mice (Fig 6D).

## Discussion

The present study determined the inhibitory activity of 15d-PGJ_2_ on breast cancer-associated bone diseases. We first investigated whether 15d-PGJ_2_ blocked breast cancer bone metastasis and the resulting bone loss. We found that 15d-PGJ_2_ attenuated cell migration and the invasion of MDA-MB-231 human breast cancer cells at noncytotoxic concentrations. Treatment with 15d-PGJ_2_ reduced the secreted levels of PTHrP through a PPARγ-independent pathway in MDA-MB-231 cells regardless of TGF-β stimulation. Cancer cell-derived PTHrP attracted attention as a key mediator of the triggering and intensification of the vicious cycle of osteolytic bone metastasis in the bone microenvironment [31]. Among the bone matrix-derived growth factors, TGF-β regulates PTHrP production via Smad-dependent or Smad-independent pathways [32–34]. Our data indicated that 15d-PGJ_2_ inhibited Smad2 phosphorylation regardless of the PPARγ pathway, and consequently, reduced levels of pSmad2 were detected in the nucleus and cytoplasm. These results suggest that treatment with 15d-PGJ_2_ suppresses the metastatic progression of MDA-MB-231 cells and decreases PTHrP production through a PPAR-independent but Smad2-dependent pathway in MDA-MB-231 cells that primarily metastasized into bone.

The majority of patients with breast cancer bone metastasis exhibit osteolytic lesions that are accompanied by severe bone destruction. Osteolysis is caused by mature osteoclasts derived from hematopoietic mononuclear precursors not a direct effect of cancer cells on bone [35]. Almost all of the other mediators that induce osteoclast differentiation and activation transduce and amplify signals through RANKL, which is produced by osteoblastic stromal cells [5]. PTHrP also enhances osteoclastogenesis and the activity of mature osteoclasts via an up-regulation of RANKL and down-regulation of its decoy receptor OPG in osteoblasts [36]. Noncytotoxic concentrations of 15d-PGJ_2_ significantly inhibited an increase in RANKL mRNA expression and a decrease in OPG mRNA expression in hFOB1.19 human osteoblastic cells stimulated with an MDA-MB-231 cell-derived conditioned medium or PTHrP in our study. Osteoclasts that are differentiated by RANKL produce acidic conditions to dissolve calcium hydroxyapatite and secrete various proteolytic enzymes, such as cathepsin K and MMPs, to degrade organic components in the bone matrix, which releases bone-stored growth factors [37,38]. Therefore, the inhibition of osteoclast-mediated bone resorption can ultimately contribute to the prevention of additional tumor growth and cancer cell-mediated osteolysis. 15d-PGJ_2_ reduced RANKL-induced osteoclast differentiation and inhibited the formation of osteoclast-mediated resorption pits by suppressing the proteolytic activities of cathepsin K and MMP-2/9. Treatment with GW9662 did not affect the anti-osteoclastogenic activity of 15d-PGJ_2_. These results indicate that 15d-PGJ_2_ blocks breast cancer-mediated bone destruction by reducing the RANKL/OPG ratio in osteoblasts that are exposed to MDA-MB-231 cell-derived osteolytic factors and inhibiting the formation and function of osteoclasts in RANKL-treated osteoclast precursors in a PPAR-independent manner.

We further estimated the inhibitory activity of 15d-PGJ_2_ on osteolytic bone metastasis in nude mice inoculated with MDA-MB-231/Luc^+^ cells into the left cardiac ventricle and subcutaneously injected with 15d-PGJ_2_ or zoledronic acid for six weeks. Metastatic progression was delayed and osteolytic lesions in mandibles, femora, and tibiae were decreased in 15d-PGJ_2_-treated mice, which was supported by bioluminescence imaging, radiographic and 3D images, and bone morphometric parameters. Moreover, the reduced serum PTHrP levels in MDA-MB-231cell-injected mice treated with15d-PGJ_2_ may be linked with the *in vitro* inhibitory effect of 15d-PGJ_2_ on PTHrP production. Zoledronic acid used as a positive control is an anti-bone resorptive agent administered to cancer patients with bone metastases at the clinical dose of 4 mg via intravenous injection every 3–4 weeks [39]. Although treatment with clinical doses of bisphosphonates (a daily dose 3 μg/kg or a weekly dose of 20 μg/kg) has been reported to inhibit skeletal tumor growth in murine models [39], zoledronic acid at high doses, 0.1 or 0.125 mg/kg, was administered to exhibit its *in vivo* anti-tumor effect in recent studies [40–42]. In this study, zoledronic acid at 0.1 mg/kg also reduced bone metastasis and bone loss in mandibles, femora, and tibiae in mice with intracardiac injection of MDA-MB-23l cells. 15d-PGJ_2_ at 2 mg/kg showed a similar effect with zoledronic acid at 0.1 mg/kg. These results demonstrate that administration of 15d-PGJ_2_ inhibits the bone metastasis of breast cancer and the resulting bone destruction.

Currently, the main clinical drugs for the treatment of cancer-associated skeletal lesions are inhibitors of osteoclastic bone resorption, including bisphosphonates and denosumab as a monoclonal antibody against RANKL [43]. These agents are beneficial for the prevention and treatment of osteoporosis in postmenopausal women [44]. In addition, bisphosphonates and denosumab decrease estrogen deficiency-related bone loss due to aromatase inhibitor therapy and cytotoxic chemotherapy in cancer patients [7]. We determined whether 15d-PGJ_2_, which exhibits potent anti-osteoclastic and anti-bone resorptive activity, prevented bone loss in OVX mice as a standard model for the pharmaceutical evaluation of estrogen deficiency-induced osteoporosis. Subcutaneously administered 15d-PGJ_2_ delayed weight gain and damage to femoral trabecular bone in OVX mice, as evidenced by bone morphometric parameters, reconstructed 3D images, histological analyses, and biochemical parameters. The serum levels of the pro-inflammatory cytokines TNF-α and IL-1β, which are key mediators of bone loss following estrogen withdrawal via the promotion of osteoclastic bone resorption [45], were also reduced in 15d-PGJ_2_-treated OVX mice. These results indicate that 15d-PGJ_2_ prevents bone loss during estrogen deficiency-inducing cancer treatment.

In summary, 15d-PGJ_2_ inhibited the proliferation, migration, and invasion of MDA-MB-231 cells and the production of a major osteolytic factor, PTHrP. 15d-PGJ_2_ also suppressed the RANKL/OPG ratio in osteoblastic cells exposed to breast cancer cell-derived osteolytic factors, the RANKL-induced differentiation of osteoclast precursors, and the formation of resorbed pits by decreasing the activities of cathepsin K and MMPs. Furthermore, subcutaneous injections of 15d-PGJ_2_ reduced the metastasis of breast cancer cells into bone and the generation of osteolytic lesions in mice. Treatment with 15d-PGJ_2_ inhibited estrogen deficiency-induced bone loss. Therefore, 15d-PGJ_2_ and 15d-PGJ_2_-inducing agents may be potent candidates for the prevention and treatment of bone loss caused by breast cancer bone metastasis and chemotherapy.
